# Discontinuation rate and associated factors among contraceptive implant users in Kersa district, southwestern Ethiopia

**DOI:** 10.1186/s13690-021-00603-6

**Published:** 2021-05-13

**Authors:** Gali Nega, Muluemebet Abera, Afework Tadele

**Affiliations:** 1 College of Medicine and Health Science, Dire Dawa University, Dire Dawa, Ethiopia; 2 Population and Family Health, Jimma University, Oromia Jimma, Ethiopia

**Keywords:** Early discontinuation, Ethiopia, Implanon, Kersa

## Abstract

**Background:**

Subdermal contraceptive implant is the most widely used method of long-acting reversible contraception among Ethiopian women. Many, however, discontinue use early, which results in unwanted pregnancies and induced abortions and their associated complications. The aim of this study is to determine the rate of and reasons for discontinuation of the contraceptive implant among users in the Kersa district of southwestern Ethiopia.

**Methods:**

A mixed-method study was carried out between March and April 2019 among 475 women who had been using the contraceptive implant between January 2015 and August 2016 in the Kersa district. Systematic random sampling was used for quantitative data collection and purposive sampling was used for qualitative data collection using 8 focus group discussion and 56 In-depth interviews. A binary logistic regression was carried out for bivariate and multivariable analyses.

**Results:**

One hundred and ten (23.2 %) contraceptive implant users requested removal before 2.5 years of use. The main reasons for the discontinuation were side effects, followed by a desire for pregnancy or to switch to another contraceptive method and misconceptions. Implant discontinuation was associated with a lack of information prior to insertion on the effectiveness of modern contraception (adjusted odds ratio [OR] 2.0; 95 % confidence interval [CI] 1.13, 3.55), being served by a midwife or nurse (adjusted OR 1.8; 95 % CI 1.04, 3.23), and not being told to return to the health facility if any side effects were experienced (adjusted OR 1.8; 95 % CI 1.01, 3.19) (all *p* < 0.05).

**Conclusions:**

Almost a quarter of the study participants discontinued use of the contraceptive implant before the due date. Public health interventions should focus on providing adequate awareness for family planning users, trainings for the health care workers on effective counselling services, especially on side effect and misconceptions.

## Background

The etonogestrel subdermal implant, implanon (Merck & Co., Inc., Canada, USA), is a highly effective method of long-acting reversible contraception (LARC) that remains effective for 3 years, making it ideal for birth spacing [1–3], and it is suitable for use in low-income countries. Its use can help reduce the high maternal mortality rate, because of its simple insertion procedure which can be provided by community-level health workers especially in rural areas. Moreover, a contraceptive implant is 120 times more effective than injectables and 180 times more effective than oral contraceptive pills [4].

The proportion of women who have an unmet need for modern contraception is highest in sub-Saharan Africa. In 2017, 43 % of the estimated 206 million pregnancies in developing regions were unintended [5]. One of the factors contributing to unintended pregnancies was contraceptive discontinuation. For instance, in Kenya and the Philippines, and in the Dominican Republic, 12 and 25 % of unintended pregnancies, respectively, were due to contraceptive discontinuation [6].

According to the 2016 Ethiopia Demographic and Health Survey, 35 % of modern contraceptive users discontinued their method before the removal date (3 years) and 11 % of contraceptive implant users discontinued the method within 12 months of use [7]. Some regions of Ethiopia, however, had a higher discontinuation rate than that found nationally. For instance, the discontinuation rate in northern Ethiopia was 65 % in Debre Tabor and 46.5 % in Debre Markos [8]. Studies have also reported different reasons for discontinuation, the main ones being wish to become pregnant, fear of side effects, and heavy or prolonged menstrual bleeding [9–12]. Owing to diverse sociocultural and socioeconomic contexts, other reasons for discontinuation were given in different parts of the country. Some rural women were not able to access free contraceptive services because of sociocultural and other unspecified barriers [11–13].

In line with global family planning targets, the Ethiopian government has updated its commitment to improve the distribution of contraception: the prevalence rate of modern contraceptive use is planned to increase to 55 % by 2020 [14]. Recent reports, however, found that in 2018, only 40.1 % of married women were using any method of contraception; of these, 23.8 % were using a contraceptive implant [15]. Despite low use of the contraceptive implant, a significant number of women request its removal before the due date (3 years) [6–9, 11, 16–19]. In Ethiopia, the discontinuation rate and associated factors have only been measured using quantitative methods. Furthermore, differences in the rates of discontinuation and factors associated with discontinuation have been reported, according to different local contexts due to the diverse ethnicities in Ethiopia. This study therefore aimed to determine the rate of contraceptive implant discontinuation and associated factors using a mixed method for qualitative data to include male responses in urban and rural areas in the Seka district of Southwestern Ethiopia.

## Methods

A community-based cross-sectional study was carried out between March and April 2019 in Kersa district, southwestern Ethiopia. Kersa district is one of 21 rural districts in Jimma Zone, Oromia Region, and is about 305 km Southwest of Addis Ababa, the capital city of Ethiopia. According to reports from the district health office, modern contraceptive coverage is 60 %, of which contraceptive implant coverage accounts for 10 % [16]. Kersa has an estimated population density of 336.8 people per square kilometer, which is greater than the Zone average of 150. The majority (88.87 %) of the inhabitants were Moslem religion followers. The study used both quantitative and qualitative methods of evaluating data collected between 1 January, 2015 and 30 August, 2016 among contraceptive implant users aged 15–49 years. Excluded from the study were women attending private clinics, women not residing in the study area during the study period and women who were unable to communicate because of illness.

### Sample size determination and sampling techniques

Epi Info, version 7.1.1, was used to determine the sample size. The calculated sample size was 475, assuming a 25 % rate of early implant discontinuation [10], a 95 % confidence interval (CI), a 5 % margin of error and a 10 % non-response rate.

Ten kebeles (small administrative wards) were selected randomly using a lottery method from 32 kebeles in Kersa district. The sampling frame was prepared for the selected kebeles by listing the registration numbers of women who started using the contraceptive implant between 1 and 2015 and 30 August 2016, taken from the family planning registers of health posts and health centers. Systematic random sampling was then used to select the study participants.

Qualitative data were collected from 8 focus group discussions (FGDs) and 7 in-depth interviews (IDIs). To control for information contamination, FGD participants were selected from diffrent randomly selected kebeles of the 22 kebeles not selected for quantitative data collection, whereas IDI participants were selected purposively from among health care providers working in the family planning units of the health centers and health posts.

### Data collection procedure

Data were collected using structured and semi-structured questionnaires that included socioeconomic, demographic, obstetric, individual, method-related, service-related and sociocultural characteristics. Face-to-face interviews were carried out to gather quantitative data, whereas qualitative data were collected by IDIs and FGDs. Discussions were held on the weekend in a school environment so that participants could feel free to express their feelings and ideas. To minimize the non-response rate, up to three attempts were made to contact women who did not respond to the invitation to participate. The recorded qualitative data were transcribed and the responses were arranged into themes to supplement the quantitative data.

### Data quality management

The questionnaire was first prepared in English and then translated into the local Afaan Oromoo language. To ensure consistency of the translation from English, the questionnaire was translated back to English by another language expert.

The questionnaire was pre-tested on 24 (5 %) women in the study area in similar population groups. Reliability test was done and Cronbach’s alpha value of 0.7 and above in each dimensions were considered for actual data collection. Four female nurses collected the data, supervised by two Public health experts having experience of community based survey, after receiving a day’s training on the study instrument and interviewing technique. All study participants were interviewed in a private place where they were able to freely express their feelings and ideas.

Data consistency and completeness were checked daily by supervisors. The FGDs and IDIs were conducted by the principal investigator. Voice recording and note taking were used to capture the information obtained from IDIs and FGDs. The results from the FGDs were summarised to supplement the quantitative data.

### Statistical analysis

The data were cleaned, coded and entered into Epidata manager, version 4.4, and exported to IBM SPSS Statistics for Windows, version 20.0 (IBM, Armonk, NY, USA), for analysis. Frequency tables, graphs and descriptive summaries were used to illustrate the study variables. Inferential statistical analysis was carried out using binary and multivariable logistic regression analysis. The Hosmer–Lemeshow test was used to assess the fitness of the models. Variables with a significance level < 0.25 on bivariable analysis, and other variables of interest, were included in the multivariable analysis. The significance of associations was set a *p*-value of < 0.05 with a 95 % CI.

The qualitative data were analysed using thematic analysis and supplemented with the quantitative data. Five themes (Benefits of implants (1 sub themes), side effects of the implants (3 sub themes), Myth and Misconceptions (6 sub themes), and health care providers related problems (1 sub themes)) with eleven subthemes were generated from the FGDs and IDIs.

### Operational definitions

- Implant refers to an Implanon which is a modern contraceptive method that is inserted subdermally in the non-dominant arm and remains effective for up to 3 years.
- Early discontinuation refers to removal of the implant before 30 months of use.
- Misconception is personal judgements without scientific evidence, the woman and/or her partner perceived that the method could cause cancer, birth complications or infertility and could migrate to another site.
- LARC refers to methods contraceptions which can prevent pregnancy for 3–10 years, but the method (i.e. implants, and copper intrauterine device) may be removed at any time beforehand if the woman wishes to conceive.
- Kebele is the smallest administrative unit below district level, with a population size of approximately 5000.
- Health extension workers are trained female community health service professionals working at kebele level on health and health-related activities.

## Results

### Sociodemographic characteristics of the study participants

Just less than half (44.8 %) of the participants were aged 25–29 years. The majority (51.2 %) were unable to read and write. Most were Muslims (92.8 %) and housewives or home workers (62.1 %) (Table 1).

**Table 1:** Sociodemographic characteristics of the 475 study participants in Kersa district, southwestern Ethiopia

Variable	*n* (%)
Age, years	
15–19	44 (9.3)
20–24	96 (20.2)
25–29	213 (44.8)
30–34	50 (10.5)
35–39	51 (10.7)
40–49	21 (4.4)
Educational level	
Unable to read and write	243 (51.2)
Able to read and write	145 (30.5)
Grades 1–4	28 (5.9)
Grades 5–8	36 (7.6)
Grades 9–12	16 (3.4)
College and above	7 (1.5)
Religion	
Orthodox Christian	28 (5.9)
Muslim	441 (92.8)
Protestant	6 (1.3)
Occupation	
Housewife/home worker	295 (62.1)
Employer	15 (3.2)
Merchant	106 (22.3)
Daily labourer	59 (12.4)

Qualitative data were acquired by interviewing two health professionals, three health extension workers, two female community health workers, in eight kebeles giving a total of 7 IDIs, and 28 women with a history of contraceptive implant use and 28 men whose wives had a history of contraceptive implant use (i.e. 56 participants in 8 FGD).

### Discontinuation rate

**Fig. 1:**
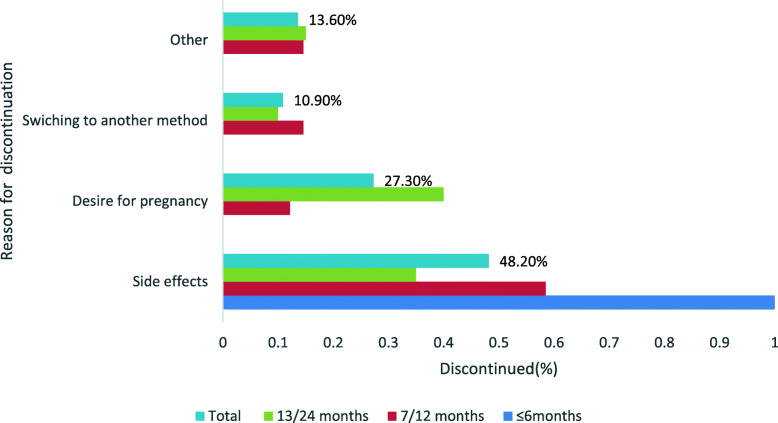
Early contraceptive implant discontinuation rate and reasons given (Kersa district, southwestern Ethiopia)

Of 475 women who had used a contraceptive implant in the last 3 years, 110 (23.2 %) had discontinued it. Discontinuation ranged from 3 to 27 months. The median duration of use was 15 months (standard deviation ± 5.8 months) (Fig. 1).

### Reasons for discontinuation

The most common reasons cited for early discontinuation were side effects and desire for pregnancy or to switch to a different method of contraception; other reasons were due to misconceptions, peer pressure or partner opposition. Side effects were the only reason (100 %) for discontinuation in the first 6 months of use and accounted for 48.2 % of all other reasons for early discontinuation during 30 months of use. One FGD participant said:

Implanon harmed my health, menstruation became longer and the amount of bleeding also increased. Moreover, the Implanon exhausted me, especially when I walked [a] long distance.

A 27-year-old female merchant.

The second most common reason for early discontinuation was the desire for pregnancy (27.3 %). However, 80 % of the women who discontinued for this reason had been using the implant for between 13 and 24 months. Some men did not support their partner’s use of the implant, because they perceived a side effect to be harmful and thought it would change their partner’s behaviour. One FGD participant said:

I knew the health problem of my wife, but now her problem is not what I knew before. She suffered from headache, dizziness and long menstrual bleeding, which was not normal.

A 34-year-old male FGD participant.

An IDI health extension worker also reported:

A client complained that she had heavy and continuous menses and loss of body weight after insertion of Implanon.

Finally, switching to another method of contraception (10.9 %) and misconceptions, peer pressure and partner opposition (13.6 %) were common reasons for early discontinuation of use. A health extension worker reported the following misconception about implant use:

A client using Implanon come to my workplace [health post] and asked me to remove it. She said that it caused cancer. She had heard on the radio that the prime minister said that Implanon caused cancer.

A 26-year-old health extension worker at Babo health post.

Some male participants believed that the contraceptive implant could have a bad effect on a woman and her fetus. One male FGD participant stated:

Implanon will store menstruation in the women’s stomach; when she gets pregnant after removal, she will suffer from pregnancy complications. She couldn’t deliver the baby because the menses that [were] stored in the stomach blocked the way that the baby comes out. Then the family took her to hospital and the doctor took out the baby from her body by surgery.

A 36-year-old male FGD participant.

Another male participant expressed similar concerns:

Let us say something about Implanon. It causes dryness of the woman’s uterus and finally causes birth complications even after they remove it and [she is pregnant].

A 27-year-old male village leader.

Partner influence was found to contribute significantly to discontinuation. One FGD participant explained why he disapproved:

We married to keep up our generation as our grandparents did, by having more children.

A 33-year-old male FGD participant.

Women who use Implanon as birth control don’t have husband interest regarding sex because the medicine that [is] found in Implanon serves as a male hormone.

A 28-year-old male FGD participant.

### Factors associated with early discontinuation

Bivariate regression analysis showed that women’s educational level, occupation, knowledge of the effectiveness of modern contraceptive methods, receipt of advice to return in the event of any side effects, place of insertion and service provider were significantly (*P*-value < 0.05) associated on bivariate regression analysis with implant discontinuation.

Multivariable logistic regression analysis showed that the service provider, the woman’s knowledge of the effectiveness of modern contraceptive methods, and being told to come back if there were any side effects were found to have a statistically significant (*P*-value < 0.05) association with discontinuation. Women who had not received information on the effectiveness of modern contraceptive methods, prior to implant insertion, were twice as likely to discontinue implant use compared with those who were given information on the effectiveness of modern contraception (adjusted odds ratio OR 2; 95 % CI 1.13, 3.55). FGD participants acknowledged the benefit of having information about modern contraception in using the implant for the full prescribed time:

I have been using Implanon repeatedly at least for more than 7 years because I know its effectiveness and I am not worried about getting pregnant.

A 37-year-old female community health worker.

The odds of early discontinuation among women who were not told to return to the health facility if they experienced any side effects were 1.8 times higher (adjusted OR 1.8; 95 % CI 1.01, 3.19) compared with those who were properly advised. Some of the qualitative study participants identified a lack of close monitoring and counselling about follow-up:

When my wife got inserted [with] Implanon at the health centre, the health workers did not tell her to go back if she faced any problems regarding the method and sometimes my wife became confused about where to seek a solution.

A 30-year-old male FGD participant.

The findings also showed that the odds of discontinuation among women who were served by a health worker were 1.8 times higher than among women served by a health extension worker (adjusted OR 1.8; 95 % CI 1.04, 3.23) (Tables 2 and 3).

**Table 2:** Qualitative study participants characteristics in Kersa district, southwestern Ethiopia

Participants	Number	Data collection method	Total
Women with a history of contraceptive implant use	28	Focus group discussion	8
Men whose wives had a history of contraceptive implant use	28		
Health professionals,	2	Indepth interviews	7
health extension workers	3		
female community health workers	2

**Table 3:** Factors associated with implant discontinuation between 1 January 2015 and 30 August 2016 in Kersa district, southwestern Ethiopia

Variable	Early discontinuation, *n* (%)		Crude OR (95% CI)	Adjusted OR (95% CI)
	Yes	No		
Educational level				
Unable to read and write	47 (19.3)	196 (80.7)	Ref.	Ref.
Able to read and write	45 (31.0)	100 (69.0)	1.87 (1.17, 3.02)***	1.80 (0.97, 3.37)
Grades 1-4	5 (17.8)	23(89.2)	0.86 (0.33, 2.51)	0.65 (0.17, 2.44)
Grades 5-8	11(30.6)	25(69.4)	1.84 (0.84, 3.99)	1.36 (0.48, 3.83)
Grades 9-12	2(8.6)	21(91.4)	0.39 (0.09, 1.75)	1.81 (0.96, 3.38)
Received information about effectiveness of modern contraception^a^				
Yes	26 (17.6)	122 (82.4)	Ref.	Ref.
No	43 (28.9)	106 (71.1)	1.90 (1.10, 3.30)***	2.00 (1.13, 3.55)*
Told to revisit health facility in case of side effects				
Yes	61 (20.1)	242 (79.9)	Ref.	Ref.
No	49 (28.5)	123 (71.5)	1.60 (1.02, 2.40)*	1.80 (1.01, 3.19)*
Service provider				
Health extension worker	45 (17.2)	216 (82.8)	Ref.	Ref.
Health worker^b^	65 (30.4)	149 (69.6)	2.10 (1.40, 3.20)***	1.80 (1.04, 3.23)*

^a^178 participants (37.5%) could not remember whether they had received this information

^b^Nurse or midwife

*p<0.05; ***p<0.001

## Discussion

### Findings and interpretation, and differences and similarities in relation to other studies

Although the contraceptive implant remains effective for 3 years, the median duration of use recorded in this study was 15 ± 5.8 months, which is less than half its expected service life. This poses a considerable challenge in addressing the high unmet need for family planning in Ethiopia.

We found an overall discontinuation rate of 23.2 % (95 % CI 19.4 %, 27.2 %), which is consistent with that found in a study conducted in Dale district, southern Ethiopia (23.4 %) [20], and in Arsi Zone, Southeast Ethiopia (25 %) [10]. It is, however, higher than found in a study from Senegal, where the overall discontinuation rate was 6.3 % [17]. This implies higher discontinuation rate of contraceptive implant in Ethiopia. The difference may be due to the educational status of the study participants, as the majority of women in our study (51.2 %) were unable to read and write. Another possible reason may be due to the variation in mean age of the participants. In addition, study participants in the current study had a younger mean age compared with those in the Senegalese study; consequently, being young is more likely to be linked to a desire for more children, which in turn leads to a higher discontinuation rate. Another reason might have been the lack of counselling during implant provision and follow-up, as 36.2 % of our study participants reported receiving no counselling about possible side effects.

In this study, we found that the discontinuation rate was 5.5 % at 6 months, 37.3 % at 1 year and 54.7 % at 2 years. These rates are higher than were found in a study in Tigray Mekele, northern Ethiopia, where the discontinuation rate at 6 months was 2.6 %, at 1 year 15.7 % and at 2 years 19.7 % [18]. The reason for this discrepancy may be the counselling that was given at the start of implant use and the subsequent continuous follow-up. For instance, in our study, 17 % of participants were counselled on possible side effects of the method and 25 % on the effectiveness of the method, whereas, in the Tigray Mekele study, 84.7 % of the participants were counselled. Therefore, counselling during insertion of contraceptive implant has a paramount impotance and should be given effectively.

Our study found that 27.3 % of discontinuations were due to a desire for pregnancy in the near future, which was probably related to the fact that the majority of participants (74.3 %) were below the age of 30 and most (74.9 %) had between one and three children. This finding accords with that of studies in South Africa [18] and Ethiopia [10, 13], in which plans to conceive in the near future were the main reason for discontinuation. Thus, providers should ensure women’s reproductive plan prior to the provision of contraceptive implants and otherwise provide appropriate family planning options.

Our study also found that there was a significant association with implant discontinuation in women who had received no information about modern contraception and had not been told to return to the health facility if they experienced any side effects. Indeed, the odds of discontinuation among women who had been given no information about modern contraception were twice those of women who had been given information. It appears that women who received clear information might have tolerated minor side effects and thus were more likely to continue with the method. Thus, appropriate ways to provide clear informations regarding modern contraceptives for all clients of contraceptive implants will improve the desired continuation rate.The main reasons given by women for discontinuation were health concerns and side effects, followed by a desire to have more children. This is consistent with the findings of another study in the Tigray region of Ethiopia [21]. Although menstrual disruption has no serious impact on women’s health, it may interfere with daily activities, especially with their sexual relationship with their husband. Some male FGD participants reported that their wife had no sexual interest. This may be a side effect of the method and the women might have been ashamed to deal with the issue with their partner if some had not first obtained their husband’s approval. Women with a lack of prior information might also have been concerned about vaginal bleeding and chosen to discontinue the method in order not to let it interfere with their sexual relationship with their husband. Other Ethiopian studies also found that inadequate counselling resulted in a high rate of discontinuation [11, 19, 22]. This implies that, providing counselling about an expected potential side effect of the method and support from service providers may be the most important way to help women continue using the contraceptive implant.

Other important predictors of early discontinuation were service-related factors. We found that women who were served by a nurse or midwife were 1.8 times more likely to discontinue early compared with those served by a health extension worker. This might have been because health extension workers are always in the community and their routine activities, especially home visiting, gave them the chance to talk to mothers and by giving information encouraged them to continue with the method without the need to revisit the health facility. In addition, women experiencing side effects might have been able to take advantage of the continuing presence of health extension workers in the community to raise complaints about the method.

Women who were not told to return to the health facility if they experienced any side effects were 1.8 times more likely to discontinue the method compared with those who received follow-up. If a complaint was made during a follow-up visit, the woman would have been given an appropriate solution by the service provider and might also have received post-insertion counselling on the expected side effects specific to the contraceptive implant. Similar findings were reported in studies carried out in Dale district in southern Ethiopia [20], Tigray in northern Ethiopia [21] and in St Louis, MO, USA [23]. Clients needs assurance from providers as a garaantee incase they expeience any side effects.

In the present study some sociocultural factors such as partner pressure and myths and misconceptions were also reported reasons for discontinuation. These may be related to a failure to provide pre-insertion counselling also to partners, as only 15.2 % of the pre-insertion counselling was couple counselling, beside partners desire for having more children. Another possible explanation is a lack of awareness and information about the method by someone in the community influencing the user. During an FGD, one woman expressed her intention to discontinue the method because she was unable to detect the implant rod and felt that the implant had moved from its original position. This could be associated with local myths and a lack of proper counselling at each service delivery point. In a qualitative study of contraceptive implant discontinuation in Butajira, southern Ethiopia, researchers found that myths, peer pressure and partner pressure were the main reasons for discontinuation [24]. Eventually, myths and miscoceptions in the community should also be resolved through different education modalities on contracptive implants inorder for the women positively influenced.

### Strengths and weaknesses

The strengths of the study were the triangulation of qualitative data, which helped to explore the local context related to early method discontinuation, and inclusion of both rural and urban participants. A limitation of the study was our inability to establish a temporal relationship between exposure and outcome variables because of the cross-sectional nature of the study design.

### Relevance of the findings: implications for clinicians and policy-makers

By providing evidence of barriers, local myths and misconceptions in the study area, to identify contextualised interventions, our findings contribute to the Ethiopian health sector plan to increase the contraceptive prevalence rate.

The provision of client information about contraceptive efficacy, and of access to contraceptive education and quality counselling services to enable desired and intended fertility, including prevention of mistimed and unwanted pregnancies, is one of the strategic focuses of Ethiopian national reproductive health policy. The study therefore contributes to knowledge development especially in the area of contraceptive implants.

We recommend working to improve communication between the district health office and community health workers and elders and other influential people in the community to reduce misconceptions related to contraceptive implant use. Furthermore, public health interventions should focus on providing adequate awareness for family planning users, trainings for the health care workers on effective counselling services, especially on side effect and misconceptions.

### Unanswered questions and future research

We recommend carrying out a longitudinal study using mixed methods to establish the causal relations of the side effects and the dynamics of contraceptive implants.

## Conclusions

Almosta quarter of the study participants discontinued contraceptive implant use within 2.5 years. Having information about modern contraception, being told to return to the clinic if there are any side effects and being served by a health extension worker were associated with the continuation of method use until the due date for removal. Experiencing a side effect, the desire for pregnancy, peer and partner pressure,myths and misconceptions were associated with method discontinuation.

## Data Availability

The data sets used are available from the corresponding author on reasonable request.

## Abbreviations

CI: Confidence interval
FGDs: Group discussions
IDIs: In-depth interviews
LARC: Long-acting reversible contraception
